# Update of the Photometric Calibration of the LASCO-C2 Coronagraph Using Stars

**DOI:** 10.1007/s11207-014-0635-2

**Published:** 2014-12-13

**Authors:** R. C. Colaninno, R. A. Howard

**Affiliations:** Space Science Division, Naval Research Laboratory, Washington, DC USA

**Keywords:** Instrumentation, Photometric calibration, Solar corona

## Abstract

We present an update to the photometric calibration of the LASCO-C2 coronagraph onboard the SOHO spacecraft. We obtained the new calibration using data from the beginning of the mission in 1996 until 2013. We re-examined the LASCO-C2 photometric calibration by comparing the past three calibrations and the present calibration with the goal of validating an in-flight calibration. We find a photometric calibration factor (PCF) that is very similar to the factor recently published in Gardès, Lamy, and Llebaria (*Solar Phys.* **283**, 667, 2013), which calculated a calibration between 1996 and 2009. The average of our PCF between 1999 and 2009 is the same, within our margin of error, as the average given by Gardès, Lamy, and Llebaria (*Solar Phys.* **283**, 667, 2013) during the same time period. However, we find a different evolution of the calibration over the lifetime of the LASCO-C2 instrument compared with past results. We find that the sensitivity of the instrument is decreasing by a constant 0.20 [±0.03] % per year. We also find no significant difference in the signal degradation before and after the SOHO interruption. We discuss the effects of this new PCF on the calibrated data set and the potential impact on scientific results derived from the previous calibration.

## Introduction

The *Large Angle Spectrometric Coronagraph* (LASCO; Brueckner *et al.*, 1995) onboard the *Solar and Heliospheric Observatory* (SOHO; Domingo, Fleck, and Poland, 1995) has been observing the solar corona almost continuously since its launch on 2 December 1995. For almost two decades, LASCO data have been the cornerstone of the study of the white-light corona. Many aspects of this study rely on an accurate photometric calibration. However, despite the importance of the calibrated data set, there has never been a consensus on the photometric calibration factor (PCF). The PCF converts the observed digital number (DN) s^−1^ pixel^−1^ into the units of mean solar brightness (MSB), the physical units traditionally used for coronagraphic data. Until recently, there have been two calibrations: the pre-flight calibration applied to the publicly available calibrated data set, and the in-flight calibration from Llebaria, Lamy, and Danjard (2006, thereafter LLD06). The published in-flight calibration has never been incorporated into the publicly available calibrated data set or included in the calibration routines available from the SolarSoft (SSW) library.^1^ A new in-flight calibration has been published by Gardès, Lamy, and Llebaria (2013, thereafter GLL13), updating the analysis through 2009. The LASCO-C2 PCF from these three sources is listed in Table 1 from data between 1996 and 2004.

**Table 1:** The LASCO-C2 PCF in units of MSB/(DN s^−1^ pix^−1^)×10^−12^; pre-flight published in the SSW library, LLD06 and GLL13 from in-flight data.

	1996	1997	1998	1999	2000	2001	2002	2003	2004
Pre-flight	6.05	6.07	6.08	6.10	6.12	6.13	6.15	6.17	6.19
LLD06	6.45	6.50	6.56	6.72	6.74	6.76	6.78	6.80	6.82
GLL13	6.96	6.97	6.99	7.24	7.26	7.29	7.31	7.34	7.36

All values are for 20 March.

The first photometric calibration of C2 was made at the Naval Research Laboratory (NRL) during assembly of the LASCO instruments. This pre-flight calibration is the basis of the PCF used in the LASCO-C2 calibration software provided to the scientific community through the SSW library. The PCF applied by the SSW calibration routines can be calculated using the equation

1 $$ C = \bigl(4.60403 \times10^{-5} \mathrm{MJD} + 3.74116\bigr) \times10^{-12}, $$

where *C* is in units of MSB/(DN s^−1^ pix^−1^) and MJD is the modified Julian date of the image. This value is returned by the SSW routine c2_calfactor.pro when given the FITS metadata of a C2 image. This routine is used to generate the calibrated (level-1) C2 data available online.^2^

**Figure 1:**
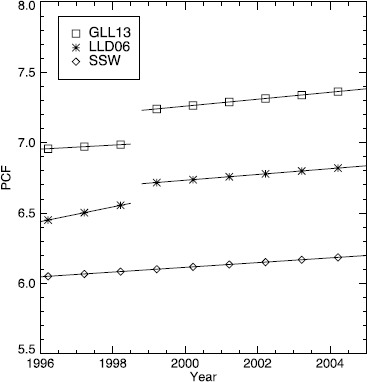
The LASCO-C2 PCF in units of MSB/(DN s^−1^ pix^−1^)×10^−12^; pre-flight published in the SSW library (diamond) and from LLD06 (asterisk) and GLL13 (square) using in-flight data.

The SSW PCF increases by 0.28 % per year; this increase was extrapolated from the degradation of the signal found by Thernisien *et al.* (2006) during the calibration of LASCO-C3. There are several possible sources for the degradation found by Thernisien *et al.* (2006), such as lens darkening due to energetic particles, changes in the filter passband or transmissivity, or changes in the quantum efficiency of the CCD. From the in-flight calibration, it is impossible to determine which elements of the C3 instrument are causing the change. However, since the CCDs in both LASCO coronagraphs are identical and both instruments have been subjected to similar environmental conditions, it was assumed that C2 would have a similar degradation in the detected signal. Thus, the annual variation found for LASCO-C3 was adopted for LASCO-C2 within the SSW routines. The pre-flight PCF for data between 1996 through 2004 is plotted in Figure 1 along with the values from LLD06 and GLL13.

LLD06 published an in-flight photometric calibration of LASCO-C2 using stars observed in the data between 1996 and 2004. LLD06 also found a small annual change in the photometry of the LASCO-C2 data. They found the best results for the calibration when they separated the C2 data into two regimes, pre- and post-SOHO interruption, which occurred between June and October 1998. Table 1 gives the PCF for 1996 to 2004 calculated using Equations (2) and (3) in LLD06. The two regimes can easily be seen in Figure 1. The annual increase in the PCF found by LLD06 is 0.81 % and 0.31 % per year for data pre- and post-SOHO interruption, respectively. The PCF published in LLD06 is greater than the SSW factor by 7 % – 10 % for 1996 to 2004.

The LLD06 calibration was not added to the SSW library routines because LLD06 did not address a fundamental aspect of the instrument operations, specifically, the time-varying CCD offset bias measured in the LASCO-C2 data. The CCD offset bias must be subtracted from the image before calculating the observed stellar flux. LLD06 also did not follow other aspects of the image pre-processing implemented in the SSW calibration routines before calibrating the images. Thus the LLD06 PCF could not be implemented within the SSW calibration routines. Consequently, all publicly available calibrated LASCO-C2 data have used the pre-flight PCF.

GLL13 published an updated in-flight photometric calibration of LASCO-C2 for the 14 years between 1996 and 2009. Their method for calibration is very similar to that used by LLD06. GLL13, however, used the correct CCD offset bias and a new method for estimating the correction for a finite aperture. GLL13 also found the best results for the calibration when the C2 data are divided by the SOHO interruption. They found an annual increase in the PCF of 0.21 % and 0.35 % per year for data pre- and post-SOHO interruption, respectively. The PCF calculated using the equations in Section 5.4 of GLL13 is listed in Table 1 for the years 1996 to 2004.

GLL13 reported excellent (< 2 %) agreement with the results of LLD06 despite the difference in the CCD bias offset used. When we compared the results from GLL13 and LLD06, we found a difference in their results of 6 % to 8 %. The difference in the PCF from GLL13 and LLD06 is apparent in Figure 1. At the scale of Figure 1, it is difficult to see the various annual change rates since they are all very similar with the exception of the pre-interruption PCF from LLD06. The values from GLL13 are 15 % – 19 % higher than the values in SSW.

In this article, we reconcile the difference between the calibration factor applied by SSW calibration routines and the published in-flight photometric calibrations. The primary goal of our work is to validate an in-flight calibration that can be used with the SSW routines and applied to the publicly available data set. We independently validate the LASCO-C2 photometric calibration using data pre-processed with the routines available in the SSW library. Thus, our PCF can be immediately implemented within the publicly available calibration routines. We focus our calibration efforts on the data generated from the C2 synoptic observations, which constitute 81 % of the total data set. We compare our new results with those found by GLL13. We discuss the effects of the new photometric calibration on scientific results derived from the calibrated LASCO-C2 data focusing on the CME mass calculations, the largest data set that relies on the photometric calibration. We then present a timeline for applying the new calibration factor to the archived and future LASCO-C2 data and adding the changes to the SSW library.

## Aperture Photometry

To perform the photometric calibration, we used stellar observations that are part of the LASCO-C2 images. Each year, the C2 field of view (FOV), from 2.2 to 7 *R*_⊙_, provides thousands of stellar observations near the ecliptic plane. We analyzed 515 stars in the C2 FOV with a minimum V-magnitude of 8.0 and a cataloged spectral type. We used the SIMBAD^3^ astronomical database values for the location, V-magnitude, and spectral type of the stars.

Similar to past in-flight calibrations, LLD06, GLL13, and Thernisien *et al.* (2006), we used aperture photometry to measure the flux from each star. As a result of the point spread function of the coronagraph optics, the flux from the stellar sources is distributed across several pixels on the CCD. With aperture photometry, the total observed stellar flux is obtained by measuring the excess brightness over the background in a region centered on the star. We did this by summing the pixel counts within a circle and subtracting the average brightness in a nearby region that accounts for the number of pixels in the circular region. We then compared these flux measurements with the cataloged magnitudes of the stars to calculate the PCF for the instrument.

To perform the aperture photometry, we used the IDL program aper.pro. This program calculates the stellar flux from the images as well as the error in the flux and the average background sky counts given the pixel location of the star. It was adapted to IDL from the Fortran package DAOPHOT (Stetson, 1987). By default, aper.pro calculates the aperture to the subpixel level by using a polygon approximation for the intersection of a circular aperture with a square pixel and normalizes the total area of the sum of the pixels to exactly match the circular area. For our measurements, we used an aperture with a radius of three pixels. To measure the value of the background sky around the star, we used an annulus with radii of four and seven pixels for the inner and outer rings, respectively.

To determine the best size of the aperture for the calibration, we used the method of Howell (1989). Howell (1989) suggested selecting the aperture for which the signal-to-noise ratio of the measurements is maximized, since as the signal increases with aperture size, so too does the contribution of the background noise. Since the background noise of coronagraphic data is high (because of the corona signal), this is a valid analytical approach to select the aperture radius. Thus, we measured the flux from several bright stars as they crossed the FOV with apertures of 1 to 7 pixel radii. Since we have multiple measurements for each star at each aperture size, we used the standard deviation of the measurements to determine the signal noise. We found that for the bright stars we analyzed, the peak signal-to-noise ratio is for an aperture radius of three pixels.

**Figure 2:**
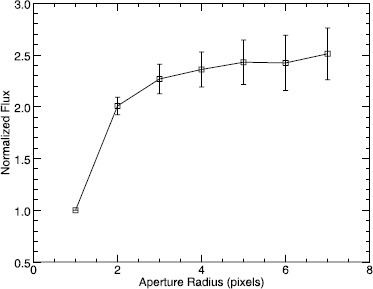
Average growth curve for stars observed by LASCO-C2. The measured stellar flux increases with aperture size along with the noise in the measurement. We chose an aperture of three pixels, which maximizes the signal-to-noise ratio. The increase in flux with increased aperture size is significantly lower than the error associated with flux.

Figure 2 shows the growth curve we constructed using measurements from several stars with increasing aperture sizes. We then took the mean of the normalized growth curves from each star. This figure clearly shows that both the measured stellar flux and the measurement error increases with aperture size. The increase in flux between an aperture of three to four pixels is an order of magnitude lower than the standard deviations of these measurements.

LLD06 used apertures with radii of 1.5, 2.5, and 3.5 pixels; the final results were based on aperture measurements of 3.5 pixels. GLL13 made measurements with radii of 1.5, 2.5, 3.5, and 4.5 pixels to calculate their growth curve. GLL13 analyzed their growth curves and calculated the correction for a finite aperture. The average change in the results for an approximated infinite aperture compared with an aperture of 3.5 pixels was only 0.04 %. We found similar results in our own fits to growth curves. Thus we conclude that the increase in flux due to a finite aperture correction is within the noise of our flux measurements and does not significantly contribute to the results.

### Image Pre-Processing

Before the observed stellar flux was measured, the LASCO-C2 data were converted to units of detected DN s^−1^. To ensure that our calibration is compatible with LASCO-C2 SSW processing routines, we used LASCO-C2 level-1 data created with SSW and divided by the calibration factor provided by the SSW program c2_calfactor.pro. The SSW processing of the LASCO images includes subtracting the CCD offset bias, applying a vignetting and geometric distortion correction, and dividing by the exposure time. As discussed earlier, the CCD offset bias of LASCO-C2 has been gradually increasing over the lifetime of the instrument. The offset bias and exposure time in the uncalibrated (level-0.5) image metadata is a best estimate of those values. The corrected values can be obtained with the SSW routine get_exp_factor.pro.

Another important aspect of the image processing before the calibration is the vignetting correction. A vignetting correction must be applied to the images, otherwise the observed flux of the stars will vary as they transit across the FOV. Because the entire FOV of C2 is vignetted (Llebaria, Lamy, and Bout, 2004), the normalization of the vignetting correction is only poorly defined. For the final calibration to be correct, the normalization of the vignetting correction and the PCF must be consistent. As with all parts of the image pre-processing, we used the vignetting correction that is available through the SSW routine. Thus the larger scientific community has access to all aspects for the image pre-processing.

One additional difficulty in applying aperture photometry to coronagraph images, as opposed to stellar images, is the bright, dynamic, and spatially variable coronal signal. In the LASCO-C2 data, the coronal signal is dominant and brighter than the stellar signal in most of the image FOV. Similarly to past coronagraph calibration methods, we have found that the aperture photometry is more accurate when applied to images where the coronal signal has been reduced. To reduce the coronal signal, LLD06 and GLL13 used a coronal model consisting of the per-pixel median of the image itself and four neighboring images, two taken immediately before and two immediately after. We found that this method for creating the coronal model included some of the stellar signal. Moreover, since this method does not take into account the actual cadence of the images, a significant amount of stellar signal was included in the background model after mid-2010 when the C2 cadence was nearly doubled. GLL13 did not examine data taken after 2009.

An important difference between this work and that of LLD06 and GLL13 is that we used a running-difference method to remove the coronal signal. We subtracted an image taken no more than 40 min after the image in which the stellar flux was measured. The temporal offset allows for the star to move across the image FOV sufficiently to not interfere with the aperture photometry measurement. This differencing method is very effective in removing the quasi-static background K-corona and the F-corona signal, but did not remove the more dynamic coronal signal (coronal mass ejections).

**Figure 3:**
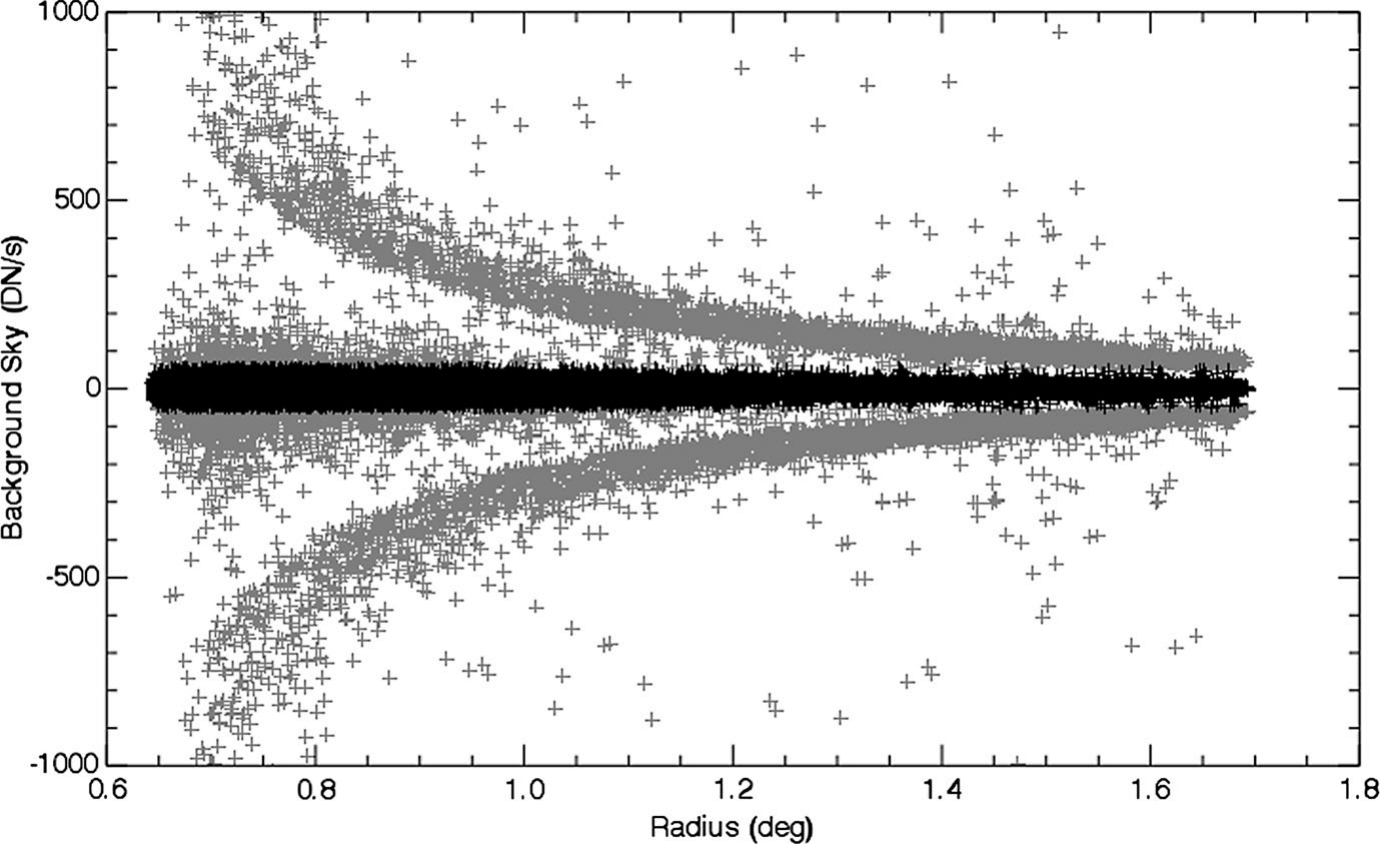
The background sky flux measured in an annulus around the star for all measurements plotted *versus* the radial distance from solar center. After the images are differenced, the background sky signal should be zero. However, the dynamic coronal signal remains in the images and appears as positive and negative *wings* in this plot. Thus, the measurements with an absolute sky signal greater than 50 DN s^−1^ (gray) are excluded from the calibration.

To analyze the effectiveness of our running-difference method, we plot the background sky *versus* radial distance from the Sun in Figure 3. The background sky is the signal measured within the annulus around each aperture of all the stellar measurements. The background sky should be near zero if the entire coronal signal has been removed. Figure 3 shows that most of the background sky measurements are distributed around zero. However, the dynamic solar activity is seen as positive and negative *wings* that decrease in intensity with radius. These positive and negative *wings* are the bright and dark K-corona features, such as coronal mass ejections, commonly seen in running difference images. Thus, we chose to exclude stellar flux measurements where the absolute background sky signal was greater than 50 DN s^−1^. The excluded measurements are plotted in gray in Figure 3. Only approximately 0.5 % of measurements were excluded by this restriction. We also excluded measurements near the occulter (radius < 1^∘^) where the noise and effects from the vignetting and dynamic coronal evolution are largest.

## Calibration Method

For each star observed by LASCO-C2, we have multiple measurements of the stellar flux during each year while the star passed through the FOV. We calculated a weighted mean flux for each star for each year. We only used stars in the annual calibration if there were more than 30 measurements within the year. On average, each mean observed brightness was calculated using 85 measurements of the stellar flux with at least 30 measurements. The weighted mean flux was calculated using the equation from Bevington and Robinson (2003),

2 $$ \bar{F}_\star= \frac{\sum_{m=1}^{n_m}{w_m F_m}}{\sum_{m=1}^{n_m}w_m}, $$

where *n*_*m*_ is the number of measurements for the star and *F*_*m*_ is the flux measurement. The weight for each flux measurement, *w*_*m*_, is defined by

3 $$ w_m = \frac{1}{\sigma^2_{f_{m}}}, $$

where $\sigma_{f_{m}}^{2}$ is the flux error derived from the aper.pro program (see Stetson, 1987). The standard deviation, *σ*_⋆_, of the mean flux, $\bar{F}_{\star}$, is given by

4 $$ \sigma_\star^2 = \frac{\sum_{m=1}^{n_m}{w_m (F_m - \bar{F}_\star )^2}}{(n_m-1) \sum_{m=1}^{n_m}w_m}. $$

This weighted average and standard deviation are the same as those used by LLD06 and GLL13.

To calculate the PCF, we compared the measured flux with the expected stellar brightness (radiative flux) in units of MSB. The expected stellar brightness cannot be taken directly from the stellar catalog because we must account for the unique passband of the C2 broadband color filters. These color filters are referred to as the blue, orange, and red filters and have a nominal bandpass of 420 – 520, 540 – 640, and 730 – 835 nm, respectively (Brueckner *et al.*, 1995). The color filter system for LASCO-C2 was designed to study the solar corona and is sufficiently different from the standard filters used for stellar photometry. The synoptic data from LASCO-C2 are taken with the orange filter. The peak of the orange filter is between that of the standard stellar B and R broadband filters (see Figure 2 in LLD06).

To calculate the expected stellar brightness in units of MSB, we used the equation

5 $$ B_\star= \frac{\Omega_\odot}{\Omega_\star} \frac{\int F_\star(\lambda) T_i(\lambda) \mathrm{QE}(\lambda)\,\mathrm{d}\lambda}{\int F_\odot(\lambda) T_i(\lambda) \mathrm{QE}(\lambda)\,\mathrm{d}\lambda}, $$

where *F*_⊙_ and *F*_⋆_ are the spectral flux of the Sun and star. *T*_*i*_ is the spectral transmission of LASCO-C2 with the *i*th filter, QE is the spectral quantum efficiency of the CCD, and Ω_⊙_ and Ω_⋆_ are the solid angles corresponding to the Sun and star, in pixels. For the stars, we assumed that they have an angular subtense equivalent to one pixel. This equation was also used in Thernisien *et al.* (2006) to calibrate the LASCO-C3 coronagraph. Each part of this equation must be calculated from experimental measurements. The spectral transmission and quantum efficiency were measured during the assembly of LASCO-C2. To calculate the spectral flux of each star *F*_⋆_, we used the data^4^ from Pickles (1998), as did LLD06 and GLL13. This catalog provides a normalized generic spectral flux profile of various star types. The Pickles (1998) catalog does not provide profiles for all stellar types. If there was no profile for the exact spectral type, we assigned the next closest type within the same series (*e.g.* G8V=G9V or G7V but G8V≠G8VI). We used the G2V spectra from Pickles (1998) for the spectral flux of the Sun, *F*_⊙_.

**Figure 4:**
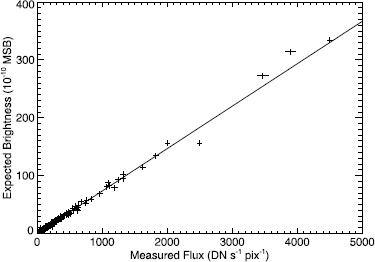
The measured flux for the 2005 observations and the expected brightness of the stars in LASCO-C2. The slope of the fitted line is the PCF in units of MSB/(DN s^−1^ pix^−1^).

We calculated the PCF directly in units of MSB/(DN s^−1^ pix^−1^) from the expected stellar brightness [Equation (5)] and the measured stellar flux [Equation (2)] using a technique similar to that used by Thernisien *et al.* (2006) for the LASCO-C3 coronagraph. Another difference between the analysis done here and LLD06 and GLL13 is that they calculated the PCF by first calculating the zero-point of the magnitudes. The zero-point is a unit-less value calculated on a log-log scale. It must be converted into PCF in units of MSB/(DN s^−1^ pix^−1^) using an equation given in LLD06 before it can be applied to the data. We calculated the PCF by plotting the mean stellar flux for each year of observations *versus* the expected brightness on a linear scale. These two quantities should be proportional. We then fit a straight line to the data; the slope of this line is the PCF. Figure 4 shows an example of these plots for the 2005 data. For each year, the error for each spectral type showed no systematic effects.

## Results

In Table 2 we list the PCF calculated for each year. In addition, we have listed the number of stars used to make this calculation. Thus the plot in Figure 4 includes 412 different observed weighted mean brightnesses as well as the standard deviations. To estimate the error in the annual PCF, we calculated the standard deviation of the slope for an exact proportionality of the two quantities. Thus the variance of the slope, $\sigma_{\mathrm{m}}^{2}$, is

6 $$ \sigma_{\mathrm{m}}^2 = \frac{\sigma^2}{\sum^n_{i=1}(x_i-\bar{x})^2}, $$

where *n* is the number of stars, and *σ* ^2^ is the variance of the fit,

7 $$ \sigma^2 = \frac{1}{n-2} \sum^n_{i=1}(y_i - \mathrm{PCF} \times x_i)^2 $$

(Bevington and Robinson, 2003). For both equations, *x* is the observed brightness and *y* is the expected brightness. We list the number of stars, the PCF, and the standard deviation of the slope in Table 2 for each year.

**Table 2:** LASCO-C2 PCF in units of MSB/(DN s^−1^ pix^−1^)×10^−12^.

	Number of stars	PCF	*σ*_m_
1996	101	6.56	0.24
1997	232	7.14	0.04
1998	263	7.37	0.07
1999	339	7.17	0.07
2000	453	7.18	0.03
2001	433	7.23	0.05
2002	455	7.32	0.05
2003	418	7.26	0.02
2004	388	7.32	0.02
2005	412	7.34	0.03
2006	419	7.38	0.05
2007	426	7.43	0.04
2008	448	7.36	0.05
2009	439	7.28	0.05
2010	460	7.41	0.04
2011	486	7.31	0.04
2012	470	7.38	0.03
2013	502	7.40	0.05

**Figure 5:**
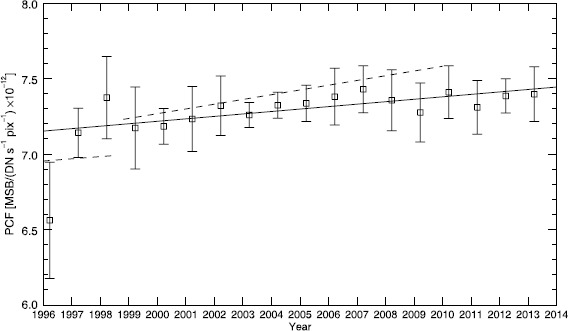
The annual variation in the calculated PCF. We have plotted the data points with 2*σ*_m_ error bars. The fit to our data is plotted with a solid line. The results from GLL06 are plotted with a dashed line.

In Figure 5, we plotted our PCF with 2*σ*_m_ error bars for each year. We fitted a linear function to these values, plotted with a solid line. We find an annual change rate in the PCF of 0.20 [±0.03] % per year for all years. We did not find a significant difference between the calibration pre- and post- interruption and thus included the data from all the years from the SOHO mission when calculating the change rate in the PCF. Thus the PCF for all dates can be found using the equation

8 $$ \mathrm{PCF} = \bigl(3.9\ [\pm0.6] \times10^{-5} \mathrm{MJD} + 5.2\ [ \pm0.3]\bigr) \times10^{-12} $$

in units of MSB/(DN s^−1^ pix^−1^). The errors on the values are the uncertainties in the slope and intercept derived from the standard deviation of the linear fit (Bevington and Robinson, 2003).

## Discussion

Even with the differences in our approaches, we find a photometric calibration factor (PCF) that is very similar to that recently published in Gardès, Lamy, and Llebaria (2013). The average of our PCF between 1999 and 2009 is the same, within our margin of error, as the average given by Gardès, Lamy, and Llebaria (2013) during the same time period. The average of our PCF between 1999 and 2009 is 7.30 [±0.08]×10^−12^; the average of GLL13 results during the same time period is 7.36×10^−12^. However, we find a different evolution of the calibration over the lifetime of the LASCO-C2 instrument compared with past results. Our annual rate of change, 0.20 [±0.03] % per year, is lower than the rate from GLL13, 0.34 % per year post-SOHO interruption. In addition, unlike LLD06 and GLL13, we did not find a significant difference in the PCF calculated for data pre- and post- interruption to justify separating the data into two regimes. In Figure 5, we plotted the PCF given in GLL13 with a dashed line. We also point out that Thernisien *et al.* (2006) did not find a change in the calibration due to the interruption, although LASCO-C3 went through similar environmental conditions.

The PCF calculated using data from 1996 has an *σ*_m_ error larger by an order of magnitude than in the remaining years. The cadence of the synoptic observations of LASCO-C2 was very different in the first years of the mission. In 1996 and 1997, LASCO-C2 observations alternated between sub-field images along the ecliptic and full FOV images. The number of images taken per day was also much lower. The difference in LASCO-C2 observations is reflected in the number of stars used to calculate the PCF each year. Table 2 shows that we used fewer stars to calculate the PCF for 1996, 1997, and 1998. To get a significant number of stars in 1996, we had to lower the minimum requirement for the number of measurements per star mean to five measurements. Thus we have larger errors for the PCF calculated before the SOHO interruption. Given these errors, we find that dividing the data into two regimes is not warranted.

We note the marked difference in the PCF for 1996 and the rest of the mission. Even though the errors are large, we wondered whether there could be a real difference in the PCF for 1996, the first year after launch. Could the instrument have quickly degraded from the pre-flight values, which are about the same as the 1996 values? To address this question, we examined the same stars as were available in the sub-field images along the ecliptic throughout the mission. In other words, instead of computing the PCF for each year for those stars, we examined the behavior for each of the individual stars during the mission and could find no trend to account for the difference in the 1996 PCF (nor in a break between 1998 and 1999). Thus we believe that there is no statistical justification to claim that the 1996 PCF is different from that of the other years. As a final point, we note that the pre-flight data had fewer observations than the post-flight data, accordingly, they had a higher statistical noise.

### Effects of Calibration Change on Quantitative Data Sets

The new PCF is 19 % – 17 % larger than the PCF currently in use. The largest data set that uses the LASCO-C2 calibrated data is the CME mass measurements in the SOHO/LASCO CME Catalog^5^ (Vourlidas *et al.*, 2000). The mass reported in the catalog is the highest value calculated for all LASCO observations of the CME. Thus, the mass reported in the catalog can be calculated using either C2 or C3 observations. We found that 55 % of the CME masses reported in the catalog are calculated with C2 data (Vourlidas, personal communications). To correct the calculated mass for the new LASCO-C2 calibration, the mass is simply increased by the percent increase of the new calibration factor.

**Figure 6:**
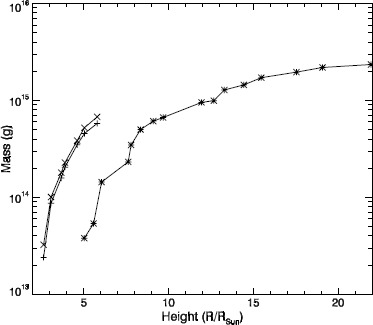
The total mass measurements from LASCO-C2 using the current calibration (plus) and the new calibration (cross), and from LASCO-C3 (asterisk) for a CME observed on 21 March 2007.

However, we find that correcting the mass is unnecessary given the large errors in this measurement. As discussed in Vourlidas *et al.* (2000), the CME masses are underestimated by about a factor of 2, for most cases. To demonstrate the assertion that correcting the CME masses is unnecessary, we present the mass measurements made in LASCO-C2 and C3 for the CME observed on 21 March 2007. These mass measurements were used in Colaninno and Vourlidas (2009) to calibrate the mass measurements made with STEREO/SECCHI-COR2. In Figure 6 we plot the total mass of the CME as calculated from LASCO-C2 images (plus) using the current calibration, and from LASCO-C3 (asterisks). We also plot the recalculated total mass of the CME in the LASCO-C2 data using the new PCF. The new masses are approximately 18 % higher than those published in Colaninno and Vourlidas (2009). The mass measurements are extremely sensitive, and we found that it was difficult to reproduce the previous values exactly before applying the new calibration. The change in the mass measurements due to the change in the calibration is negligible compared to the error in the mass measurements.

### Implementation of Changes

NRL maintains a database of calibrated (level-1) LASCO-C2 data that is accessible to the public through the LASCO website.^6^ This dataset will be updated to include the photometric calibration presented in this article. In addition, all calibration software provided through SSW will also be updated to reflect the new photometric calibration. All changes to the C2 level-1 dataset will occur on a date published on the LASCO website

## Footnotes

1
http://www.lmsal.com/solarsoft/.

2
http://lasco-www.nrl.navy.mil/.

3
http://simbad.u-strasbg.fr/simbad/.

4
http://www.ifa.hawaii.edu/users/pickles/AJP/hilib.html.

5
http://cdaw.gsfc.nasa.gov/CME_list/.

6
http://lasco-www.nrl.navy.mil/.
